# Exploring Barriers and Bridging Gaps: Perspectives of Nurses, Midwives, and Lady Health Workers in the Implementation of Emergency Obstetric Care

**DOI:** 10.12669/pjms.41.1.10269

**Published:** 2025-01

**Authors:** Fouzia Gul, Razia Mehsood, Shehla Noor

**Affiliations:** 1 Prof. Dr. Fouzia Gul, Department of obstetrics & Gynaecology, Khyber Medical University Institute of Medical Sciences, Liaqat Memorial Hospital, Kohat, Pakistan; 2 Dr. Razia Mehsood, Associate Professor, Department of obstetrics & Gynaecology, Khyber Medical University Institute of Medical Sciences, Liaqat memorial hospital Kohat, Pakistan; 3 Prof. Dr. Shehla Noor, Department of obstetrics & Gynaecology, Ayyub Medical College, Abbottabad, Pakistan

**Keywords:** Emergency obstetric care (EmOC), Health care providers, Challenges, Barriers, Maternal mortality, Nurses, Lady Health Visitors, and Lady Health Workers

## Abstract

**Background & Objectives:**

Maternal mortality is a global concern primarily due to preventable obstetric complications. Challenges in implementing Emergency Obstetric Care (EmOC) in developing nations hinder effective reduction of these deaths. Our objective was to identify key challenges in EmOC practices among frontline healthcare providers, assess the severity and frequency of these barriers, and evaluate gaps in resources, training, and institutional support needed for effective resolution.

**Methods:**

This cross-sectional study assessed EmOC knowledge, practical skills, and challenges among Nurses, Lady Health Visitors (LHVs), and Lady Health Workers (LHWs) in Khyber Pakhtunkhwa, Pakistan from July 2017-June 2020. Participants were conveniently sampled from hospitals across eight districts, with data collected through a questionnaire on EmOC knowledge, practical interventions, and challenges faced.

**Results:**

Although most participants (91% of nurses, 90.2% of LHVs, 92.3% of LHWs, and 83.3% of midwives) expressed satisfaction with course content, important gaps emerged in the practical application of specific EmOC interventions. Nurses displayed 79% knowledge of the Partogram with only 60% utilization, while LHVs showed 80.5% knowledge but just 50.2% utilization. Assisted delivery knowledge and practice were particularly low among nurses (32% & 7%) & LHVs (17.1% & 2.4%) respectively. Significant gaps were found in retained placenta removal and manual vacuum aspiration, with only 12% of nurses and 3% of LHVs practicing the latter, with no utilization among LHWs or midwives. Key barriers included insufficient training and restrictive institutional policies.

**Conclusion:**

Despite high course content satisfaction, disparities in EmOC knowledge and practice persist among frontline healthcare-providers, with barriers like insufficient training and institutional limitations impacting maternal outcomes.

## INTRODUCTION

About 70% of maternal deaths results from direct obstetric complications like haemorrhage, infection, eclampsia, prolonged or obstructed labour, complications of abortion, ectopic pregnancy and ruptured uterus^1^ Most of these complications cannot be anticipated and may occur unpredictably in otherwise healthy women who have received good antenatal and/or intrapartum care.^2^ However nearly all of these maternal fatalities can be prevented through suitable and prompt identification and treatment measures.

Realizing globally high maternal mortality, the World Health Organization (WHO) took an initiative to reduce maternal mortality by providing greater access to health care through Emergency Obstetric Care (EmOC) program worldwide.^3^ This initiative was primarily focused on low and middle-income countries.^4^ EmOC refers to ‘care provided in health facilities to treat direct obstetric emergencies that cause the vast majority of maternal deaths during pregnancy, at delivery and during the postpartum period’.^5^ EmOC comprises two components: *Basic EmOC services*, such as administering antibiotics, anticonvulsants, uterotonic drugs, manual removal of the placenta, assisted vaginal delivery, and removal of retained products; and *Comprehensive EmOC*, which encompasses all basic services along with capabilities for blood transfusion and Cesarean sections, offering a broader spectrum of essential maternal care.^6^

Numerous studies affirm the crucial role of EmOC as an integrated package within healthcare programs to effectively diminish maternal mortality.^7^ EmOC services serve as valuable indicators for evaluating health system performance, with variations in their quality quickly reflected in measurable outcomes, particularly in terms of maternal and infant mortality.^8^

Despite huge global efforts, the expected reduction in maternal mortality of 75% by 2015 was not achieved. Maternal mortality is still a major global issue with 216 deaths per 100,000 live births^9^ & approximately 830 maternal deaths around the world per day.^10^ Pakistan holds one of the highest maternal mortality rates in the South Asian region, as highlighted by recent data.^11^ In 2019, the reported maternal mortality ratio (MMR) for Pakistan was 186 deaths per 100,000 live births, reflecting a notable 32% surge from the 2017 figure of 140 deaths per 100,000 live births.^12^ It’s worth noting that there is a substantial discrepancy between rural and urban areas. The rural regions exhibit a significantly higher MMR of 199 deaths per 100,000 live births, compared to the urban areas, which report an MMR of 158 deaths per 100,000 live births.^12^ Despite Pakistan’s commitment to the Agenda 2030 and the pursuit of Sustainable Development Goals (SDGs), there remains a considerable gap between aspiration and achievement in this domain.^11^ This target entails reducing the MMR to fewer than 70 deaths per 100,000 live births by the year 2030.^13^

Despite global efforts such as EmOC program, Pakistan continues to experience a high incidence of maternal deaths, indicating a gap between expected and realized outcomes. To bridge this gap, there is a pressing need to explore the challenges faced by frontline healthcare providers in implementing EmOC. Understanding these barriers is essential for developing targeted interventions and improving the delivery of maternal healthcare services, ultimately contributing to the reduction of maternal mortality rates in Pakistan and similar settings. This study was planned to identify and categorize key challenges impacting EmOC practices among frontline healthcare providers—including nurses, lady health visitors (LHVs), lady health workers (LHWs), and midwives—while assessing the severity and frequency of these barriers and evaluating current versus required resources, training, and institutional support needed to address them effectively.

## METHODS

This cross-sectional study was conducted on a diverse sample of participants (nurses, LHVs & LHWs) working in various categories hospitals in eight districts of KPK from July 2017-June 2020. Data pertaining to doctors’ perspectives on EmOC, which is a component of our broader research project, is currently undergoing publication in another journal.

### Ethical Approval:

The study was approved by the Research Ethics Committee of Khyber Medical University Institute of Medical Sciences, Kohat under reference number 40/KIMS/IRBB/RP/2016, dated 23-08-2016.

The various randomly selected districts were Kohat, Karak, Hangu, Bannu, Dera Ismail Khan, Peshawar, Charsada, & Abbottabad. The questionnaire was designed to cover all relevant aspects of EmOC knowledge and practical application to get comprehensive data on the participants’ understanding and experiences with EmOC. The questionnaire had three parts. The first part had questions related to the basic knowledge of participants regarding EmOC. The second part had questions how they practically applied their knowledge in carrying out various lifesaving interventions & third part was about what practical difficulties they faced during implementation of EmOC interventions. Convenient sampling technique was used for data collection. Questionnaire were distributed among study participant via mail & WhatsApp. The quarries, concerns or doubts of participants regarding any parameter were addressed through telephonic contact to get in-depth information.

Data was analysed using IBM SPSS Statistics version 24.0. Chi-square test was used to evaluate the associations between various categorical variables, including the hospital-wise distribution of healthcare workers, comparative analysis of EmOC interventions, discrepancies in knowledge and practical application, and challenges faced by healthcare providers in implementing EmOC practices, to identify any statistically significant differences.

## RESULTS

Out of 160 healthcare workers, 100 (62.5%) were nurses, 41 (25.6%) were LHVs, 13 (8.1%) were LHWs and 6 (3.8%) were midwives. Majority of nurses were having basic qualification of FA (44%) & BA (38%) while LHVS 22 (53.7%), LHW 12 (92.3%) & midwives 3 (50%) were matriculate. The hospital wise distribution of theses health care workers has been shown in Table-I.

**Table-I:** Hospital wise distribution of health care workers.

Hospitals	Nurses N=100	LHVs N=41	LHWs N=13	Midwives N=6
Tertiary Care	41 (41%)	3 (7.3%)	0	0
District headquarter hospital	56 (56%)	24 (58.5 %)	4 (30.8%)	5 (83.3%)
Tehsil headquarter hospital	2 (2%)	8 (19.5%)	0	0
Rural health centre	1 (1%)	5 (12.2%)	3 (23.1%)	1 (16.7%)
Basic health unit	0 (%)	1 (2.4%)	6 (46.2%)	0

The knowledge of our frontline healthcare workers about various components of emergency obstetric care services was very poor. Only 33.7% of the participant were able to give correct answer that EmOC has two main functional components.

When knowledge about various signal functions of EmOC was compared among various cadres of health care professionals, the reported percentages highlight significant variations for EmOC interventions across the various healthcare professional groups as is obvious from Table-II.

**Table-II:** Comparative analysis of emergency obstetric care interventions among different healthcare workers.

Interventions	Nurses N=100	LHV N=41	LHW N= 13	Midwife N=6	P-Value
IV Antibiotics	25 (25%)	3 (7.3%)	0 %	1(16.7%)	.000
I/V oxytocin	39 (39 %)	3 (7.3%)	0 %	1(16.7%)	.000
Anticonvulsants	16 (16%)	4 (9.8%)	0 %	0 %	.000
Manual placenta removal	46 (46%)	4(9.8%)	0 %	0 %	.000
RPOCS	40 (40%)	3 (7.3%)	0 %	0 %	.000
Assisted delivery	43 (43%)	3 (7.3%)	0 %	0 %	.000
Blood transfusion	34 (34%)	2 (4.9%)	0 %	0 %	.000
c- section	26 (26%)	1 (2.4%)	0 %	0 %	.000

RPOCS: Retained products of conception.

The statistically significant differences emphasize the importance of targeted training and interventions to ensure consistent and appropriate emergency obstetric care practices among all healthcare providers.

Though majority of health workers were satisfied with course content nurses 91 (91%), 37 (90.2%) LHV, 12 (92.3%) LHW & 5 (83.3%) midwives, but statistically significant discrepancy in knowledge & practical use of partogram, Vaccume/forceps delivery, manual vacuum aspiration (MVA) use, manual removal of retained placenta was observed, (Table-III).

**Table-III:** Discrepancy in knowledge & practical use of various interventions.

	Nurses N=100	LHVs N=41	LHWs N=13	Midwives N=6	P-Value
Partogram knowledge	60 (60%)	21 (50.2%)	1 (7.7%)	4 (66.7%)	.001
Partogram use	79 (79%)	33 (80.5%)	4 (30.8%)	4 (66.7%)	.001
Assisted delivery knowledge	32 (32%)	7 (17.1%)	3 (23.1%)	0 (0 %)	.000
Assisted delivery practice	7 (7%)	1 (2.4%)	1 (2.4%)	0 (0 %)	.000
Retained placental removal knowledge	68 (68%)	23(56.1%)	5 (38.5%)	2 (33.3%)	.001
Retained placenta removal practice	51 (51%)	15(36.6%)	4 (30.8%)	2 (33.3%)	.002
MVA use knowledge	41 (41%)	18(43.9%)	4(30.8%)	0 (0 %)	.005
MVA use practice	12(12%)	3(7.3%)	2 (33.3%)	0 (0 %)	.005

LHVs: Lady Health Visitors, LHWs: Lady Health Workers, MVA: manual vacuum aspiration.

The primary factors identified as contributing to the limited practical implementation of these skills were either inadequate training in these areas or being prohibited from carrying out these interventions by the respective hospital authorities. This is obvious from Table-IV.

**Table-IV:** Implementation challenges and restrictions for various emergency obstetric care practices among different healthcare providers.

EmOC practices		Nurses N=100	LHVs N=41	LHWs N=13	Midwives N=6	P-value
Placental removal	Not allowed	32 (32%)	2 (4.9%)	2 (15.4%)	2(33.3%)	.002
	Cannot perform	17 (17%)	16 (39%)	7 (53.8%)	2(33.3%)	
Partogram	Not available	31 (31%)	15(36.6%)	2 (15.4%)	3 (50%)	.05
	Cannot use	36 (36%)	20(48.8%)	10(76.9%)	2 (33.4%)	
E &C	Not allowed	48(48%)	10(24.4%)	9(69.2%)	1(16.7%)	.005
	Cannot perform	22(22%)	15(36.6%)	2 (15.4%)	2 (33.3%)	
Assisted Delivery	Not available	2 (33.3%)	7 (17.1%)	9 (69.2%)	2 (33.3%)	.01
	Cannot use	46 (46%)	29(70.7%)	0 (0%)	0 (0%)	

LHVs: Lady Health Visitors, LHWs: Lady Health Workers, E & C: evacuation and curettage, EmOC: Emergency Obstetric Care Practices.

The use of MVA was the poorest skill being clinically practiced, only by 12 (12%) nurses, 3 (7.3%) LHV & none of LHW & midwives were practicing it. Various obstacles identified by health care providers towards successful implementation of EmOC interventions are given Table-V.

**Table-V:** Challenges encountered by healthcare providers in emergency obstetric care implementation.

	Nurse	LHV	LHW	Midwife	P-value
Distant residence	55(55%)	22 (53.7%)	10(76.9%)	4 (66.7%)	0.44
Travelling distance >30 minutes	55(55%)	24 (58.6%)	9(69.3%)	3(50%)	0.685
Poor pay scale	42(42%)	21 (51.2%)	9 (69.25)	6(100%0	0.02
Difficulty in night shift duty	72(72%)	28(68.3%)	11(84.6%)	2(33.3%)	.05

## DISCUSSION

This study significantly enhances the understanding of EmOC implementation challenges in Khyber Pakhtunkhwa, Pakistan, through quantitative assessment of key EmOC intervention knowledge and utilization among nurses, LHVs, and LHWs. Although satisfaction with the course content was generally high among all groups, significant disparities were evident between theoretical knowledge and practical application, specifically concerning tools like the Partogram and procedures such as MVA. The findings highlight specific challenges such as inadequate training and restrictive hospital policies, which hinder effective EmOC delivery. Additional factors contributing to the poor implementation of EmOC included inadequate salary packages, long distances from hospitals to residences, and domestic responsibilities, particularly night shift duties were challenging for healthcare workers.

Training of emergency obstetrics and gynaecology plays a pivotal part in increasing quality of care and reducing maternal and perinatal mortality and morbidity. In our study though more than 90% participants were satisfied with course content & knowledge being disseminated but the practical application of knowledge & skills was not satisfactory as obvious from Table-III. In certain parts of the world, still the health care providers lack basic knowledge & essential health care skills.^14^ The main obstacle in proper implementation of EmOC services in low & middle income countries (LMIC) was employment of inappropriately selected lectures & trainers, who themselves were deficient in knowledge & practical skills being required for EmOC practices. 15,16 This led to lack of competency among nurses & LHV & poor quality of EmOC service delivery.^7^,^8^ Same discrepancies in knowledge & practice of EmOC services were observed in a study conducted in Ethopia.^8^ A similar gap in practical skills within the existing curriculum was identified by LHWs when implementing community-based interventions outlined in the Essential Package of Health Services by the government of Pakistan.^17^

Insufficient income opportunities, and compromised living standards can lead to decreased productivity and efficiency, as well as a higher likelihood of burnout and dissatisfaction among employees. More than 50% of our study participants showed dissatisfaction with their salary package. Same economic issues in the form of low salaries, infrequent payments & a lack of governmental financial commitment have been identified as the most common barrier in successful implementation of EmOC services.^18^-^20^

The various barriers faced by pregnant ladies to access health care facility has been described in the form of “three delays” model but there is no such model on barriers by the women who are providing emergency health care.^21^ Women providing midwifery care were stigmatized as “uneducated women of doubtful moral character”, with no respect in society.^19^ Our study also showed that 53.8% of study participant expressed various domestic reason as barrier to provision of emergency services, 21.3% were unable to cope because of distant residence & 25.1% were facing difficulty in night duty because of prolonged shift of 12 hours & heavy workload during night shift. A study done in Afghanistan showed male dominant cultural barrier as one of obstacle towards successful implementation of health services by female health workers.^18^

Nursing & midwifery personnel in LMICs face the challenges of their domestic and reproductive responsibilities with their professional low paid duties.^22^ The specific demands of the midwifery role, with excessive working hours outside the home, especially at night, can strain relationships and create a sense of insecurity within the marriage. These various factors of being overburdened with prolonged duty hours have also been reported as one of the reasons of poor EmOC service. A study done in Tanzania also showed inconsistent provision of EmOC signal functions mainly because of policy restrictions, lack of supplies and professional development.^23^

Lack of proper teaching & training resulted in poor skills. Low salary packages & various domestic & cultural barriers along with poor skills resulted in lack of confidence in this frontline health work force which in turn led to failure in achieving target reduction in maternal mortality.

This study, the first of its kind in Pakistan, provides critical insights into the challenges of implementing EmOC across a diverse sample of healthcare professionals—including nurses, LHVs, LHWs, and midwives—from central, southern, and northern districts of Khyber Pakhtunkhwa province of Pakistan. It assessed EmOC knowledge, practical skills, and implementation barriers, revealing key differences among provider categories. The findings identify significant gaps between theoretical knowledge and practical application, particularly due to insufficient training and restrictive hospital policies, highlighting the need for targeted interventions and policy reforms. By capturing a broad spectrum of perspectives across various healthcare facility levels, from tertiary hospitals to BHUs, this study provides valuable data to inform efforts that aim to strengthen maternal healthcare outcomes in low-resource settings globally.

### Limitations:

The study utilized convenient sampling, resulting in an uneven distribution of healthcare worker categories and an absence of precise sample size calculation. This sampling approach was selected due to logistical challenges in reaching certain groups. Additionally, the reliance on self-reported data could introduce bias, potentially affecting the generalizability of the findings, especially for midwives and LHWs.

## CONCLUSION

This study highlights critical disparities in EmOC knowledge and practical skills among nurses, LHVs, LHWs, and midwives across eight districts of Khyber Pakhtunkhwa, Pakistan. Although most participants were satisfied with course content, only 33.7% accurately identified essential EmOC components, with inconsistent practical application of interventions like partogram use, MVA, and assisted deliveries. Contributing factors include inadequate training, restrictive hospital policies, and socio-economic barriers, such as low salaries and domestic responsibilities. These findings emphasize the need for focused training, policy reforms, and systemic support to enhance healthcare provider competency in low-resource settings. Targeted interventions to bridge these gaps are essential for advancing maternal health outcomes and reducing maternal and perinatal mortality. This study provides actionable insights for policymakers and healthcare institutions to strengthen EmOC delivery in Pakistan.

### Recommendations:

Future research should focus on larger, more representative samples to improve generalizability, especially for midwives and LHWs. Longitudinal studies could assess the impact of EmOC training on outcomes, while qualitative research may uncover personal and systemic challenges in EmOC delivery. Investigating policy and hospital support effects on EmOC practices can also help inform strategies to strengthen maternal health services in low-resource settings.

### Authors’ Contribution:

***Fouzia Gul:*** Cconcept & design, acquisition of data, analysis & interpretation, Drafting.

**Razia Mehsood: A**cquisition of data, Critical revision,

**Shehla Noor:** acquisition of data, critical analysis.

All authors have read the final version to be published and are accountable for integrity of the study.
